# The comparison of lesion outline and temperature field determined by different ways in atrial radiofrequency ablation

**DOI:** 10.1186/s12938-016-0251-5

**Published:** 2016-12-28

**Authors:** Zhen Tian, Qun Nan, Xiaohui Nie, Tong Dong, Ruirui Wang

**Affiliations:** College of Life Science and Biomedical Engineering, Beijing University of Technology, No. 100 Pingleyuan, Chaoyang District, Beijing, China

**Keywords:** Atrial fibrillation, Radiofrequency ablation, Finite element method, Thermal dosage, Hyperbolic equation

## Abstract

**Background:**

The aim of this study is to research the lesion outline and temperature field in different ways in atrial radiofrequency ablation by using finite element method.

**Methods:**

This study used the method which considered the thermal dosage to determine the boundary between viable and dead tissue, and compared to the 50 °C isotherm results in analyzing lesion outline. Besides, we used Hyperbolic equation which considered the relaxation time to calculate the temperature field and contrasted it with Pennes’ bioheat transfer equation.

**Results:**

As the result of the comparison of the lesion outline, when the ablation time was 120 s, the isotherm of the thermal dosage was larger than the 50 °C isotherm and with the increasing of the voltage the gap increased. When the ablation voltage was 30 V, the 50 °C isotherm was larger than the thermal dosage isotherm when the ablation time was less than 160 s. The isotherms overlapped when the time was 160 s. And when the ablation time was more than 160 s, the 50 °C isotherm was less than the thermal dosage isotherm. As to the temperature field, when the ablation voltage was 30 V with the ablation time 120 s the highest temperature decided by Hyperbolic was 0.761 °C higher. The highest temperature changed with relaxation time. In most cases, the highest temperature of the Hyperbolic was higher otherwise the relaxation time was 30–40 s.

**Conclusions:**

It is better to use CEM43 °C to estimate the lesion outline when the ablative time within 160 s. For temperature distribution, the Hyperbolic reflects the influence of heat transmission speed, so the result is more close to the actual situation.

## Background

Atrial fibrillation (AF) is the most common arrhythmia cardiac symptoms, the incidence of it increases with age. It also contacts with some other diseases, such as stroke and heart failure which can degrade the quality of life and increase rates of death [1]. Anti-arrhythmic drugs and surgical operation are the two major therapeutic methods in restoring and maintaining sinus rhythm. However, the incompletely effective and the dangerous side effects of the anti-arrhythmic drugs limit their long-term use. Also, the complexity of the surgical operation limit the popularization of this method [2]. While radiofrequency ablation (RFA) which used electric current to cut the accessory pathways of abnormal tissue and targeted at certain points which produce cardiac arrhythmia. In recent years, due to its advantages of safety, minimally invasive and so on, it was widely used in treatment atrial fibrillation [3].

In the process of RFA the electromagnetic energy is converted to heat. Tissue temperature above 50 °C are reserved for direct treatment, and the therapy is termed ablation [4, 5]. Dewhirst found that the cell survival/CEM43 relationship closely aligns with isothermal exposure of tissue to temperatures of 50 °C [6]. During RFA 50 °C isotherm which only consider temperature is regarded as the boundary of necrotic cells and survival cells [7–11]. However, many studies show tissue damage is concerned with both temperature and time. On the basis of the conception, thermal dosage which consider both temperature and time was put forward. 43 °C is used as the benchmark temperature how long the tissue to absorb heat in order to keep 43 °C is expressed as cumulative equivalent minutes at 43 °C (CEM43 °C) [11]. So this study created finite element method (FEM) models of cardiac and determined the temperature field in the tissue solving by the Pennes’ bioheat transfer equation [12, 13]. And comparison of the lesion range which determined by the 50 °C isotherm with the lesion range which determined by thermal dosage.

In most of the simulations, Pennes’ bioheat transfer equation which is based on Fourier’s heat theory determined the temperature field. But Fourier’s heat conduction theory is the law of macro-continuity. This theory does not have time item, which implies the speed of heat is infinite. So it also applies in the immediate energy diffusion at the infinite propagation speed in the medium. In most situations, Pennes’ bioheat transfer equation can meet the conditions, but when involved in very low or high temperature, very high heat flux or very short heating duration Fourier’s heat conduction theory breaks down. The reason is the wave nature of heating processes becomes pronounced [14–19]. Hyperbolic equation is based on No-Fourier’s heat theory which thinks about heat wave propaganda speed (relaxation time). In order to consider the condition of the short heat duration time, this studies took Hyperbolic equation into account and contrasted the temperature field which determined by the two equations.

This paper uses the finite element method of cardiac radiofrequency ablation to determine the damage area size. The simulation research, comprehensive analysis of the differences of the temperature field determined by both thermal dose method and 50 °C isotherm. The thermodynamics equation Pennes’ bioheat transfer equation and Hyperbolic equation was also considered. The model was referenced in the paper [20].

## Methods

### Geometric model

**Fig. 1:**
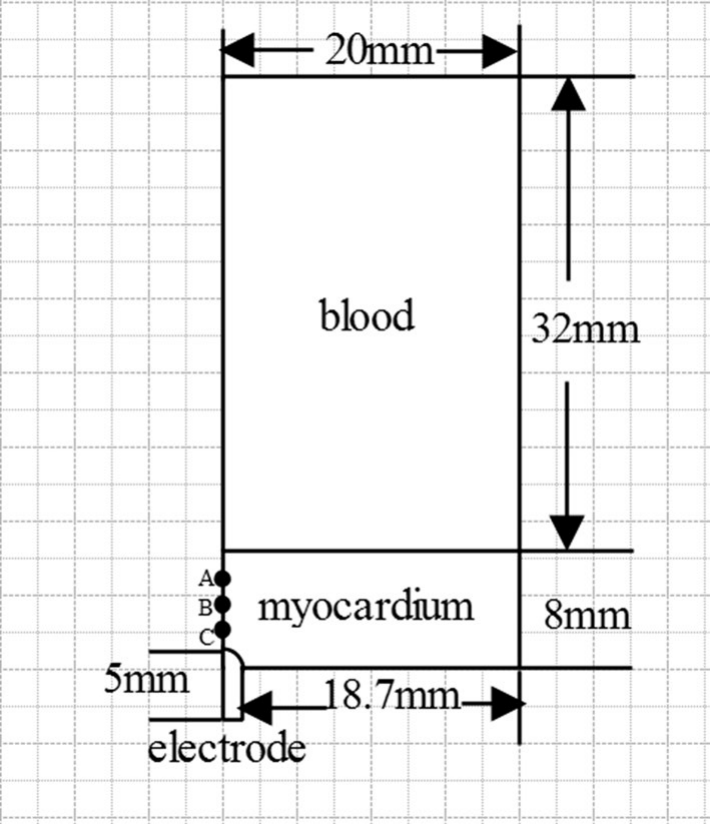
Cardiac model

The idealized 2D axisymmetric simplified model of cardiac radiofrequency ablation was shown in Fig. 1 (Cardiac model) which included myocardium, myocardial coverage (usually identified as blood) and electrode. We used the finite element method (FEM) software Comsol Multiphysics to solve partial differential equations of single field and multi-field in order to achieve the numerical results. As shown in Table 1 [21, 22], the thermal and electrical parameters of myocardium, blood and radiofrequency electrode were added to the related materials. These values are derived from physical experiments, and have been cited by others [23–25].

**Table 1:** The thermal and electrical parameters [21, 22]

	Density (kg/m^3^)	Thermal conductivity (W/(m °C))	Conductivity (S/m)	Specific heat capacity (J/(kg· °C))
Myocardium	1200	0.55	0.222	3200
Blood	1000	0.543	0.667	4180
Radiofrequency electrode	21,500	71	4,000,000	132

**Fig. 2:**
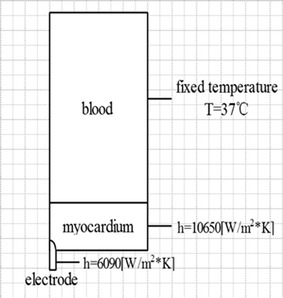
Boundary condition for analysis

The voltage was set at the top of the electrode. The convection coefficient in surface of the electrode was 6090 W/(m^2^ K), the convection coefficient of the inner surface of the heart was 10,650 W/(m^2^ K) [20]. The initial temperature was 37 °C. The boundary of this model showed in Fig. 2 (boundary condition for analysis).Mesh refined near the radiofrequency electrode automatically. Mesh independence analysis was accomplished to affirm the accuracy and it’s reliable to the simulation results for ablation processes.

### Electromagnetic and Pennes’ bioheat transfer equation

COMSOL provides the electromagnetic field module and heat transfer module. The formulas of both physical fields are as follows.

1 $$\nabla \cdot J = Q_{r}$$

2 $${\text{J}} = \left( {\upsigma + \varepsilon_{0} \varepsilon_{\gamma } \frac{\partial }{\partial t} } \right){\text{E}} + J_{e}$$

where σ is the electrical conductivity, $\varepsilon_{0}$ and $\varepsilon_{r}$ are the permittivity of tissue respectively, E is the electric field intensity, and J is the electric current density.

In the first situation the temperature distribution in the cardiac model was obtained by solving the Pennes’ bioheat transfer equation [12, 26–28].

3 $$\uprho {\text{c}}\frac{\partial T}{\partial t} = k\nabla^{2} {\text{T}} + \omega_{b} c_{b} \left( {T_{b} - T} \right) + Q$$

4 $${\text{Q}} = Q_{m} + Q_{r}$$

where $\rho$ is tissue density (kg/m^3^), c is specific heat of tissue (J/kg °C), k is the thermal conductivity (W/m °C), T is the temperature, Qr is the heat source(W/m^3^), and Q_m_ is the perfusion heat loss. In this simulation we didn’t take the Q_m_ into account for its slight effect compared with the large blood vessel.

In the other situation, the temperature distribution in the cardiac model was obtained by solving the Hyperbolic equation which considered relaxation time [17].

### Definition of the boundary between the viable and dead tissue

According to the temperature distribution, there are two main transformation algorithms to estimate the ablation region, and to calculate the transverse width, longitudinal length, and the area of the lesion. In this model, we used 50 °C isotherm and CEM43 °C to estimate the thermal damage region.

For tissue temperature above 50 °C are reserved for direct treatment, and the therapy is termed ablation [4, 5]. Dewhirst found that the cell survival/CEM43 relationship closely aligns with isothermal exposure of tissue to temperatures of 50 °C [6]. In RFA, 50 °C is regarded as the boundary of necrotic cells and survival cells, and it is easy and convenient for 50 °C isotherm threshold. So in this model, we use it to analyze.

Cumulative equivalent minutes of thermal treatment at 43 °C (CEM43 °C) method [29] take the temperature history into account, and it commonly used in the hyperthermia. The calculation formula is as follows:

5 $$\mathop \sum \limits_{t = 0}^{t = end} R^{{(43 - T_{\Delta t} )}} *\Delta t$$

where t is time (s),T_Δt_ is the average temperature during time Δ*t* (°C), Δ*t* is time interval (s). R is a parameter when temperature above 43 °C is 0.5 while the temperature below 43 °C is 0.25 [30].

As there is no critical CEM43 °C for cardiac tissue, we estimate CEM43 °C for myocardium to be 128 min (7680 s) to analyze [8].

## Results

The comparison of the lesion outline decided by the CEM43 °C and decided by 50 °C isotherm were selected for result illustration. In addition, the distribution of temperature field calculated by the Hyperbolic equation and the Pennes’ bioheat transfer equation were both researched.

### The comparison of the lesion outline in different voltages

**Fig. 3:**
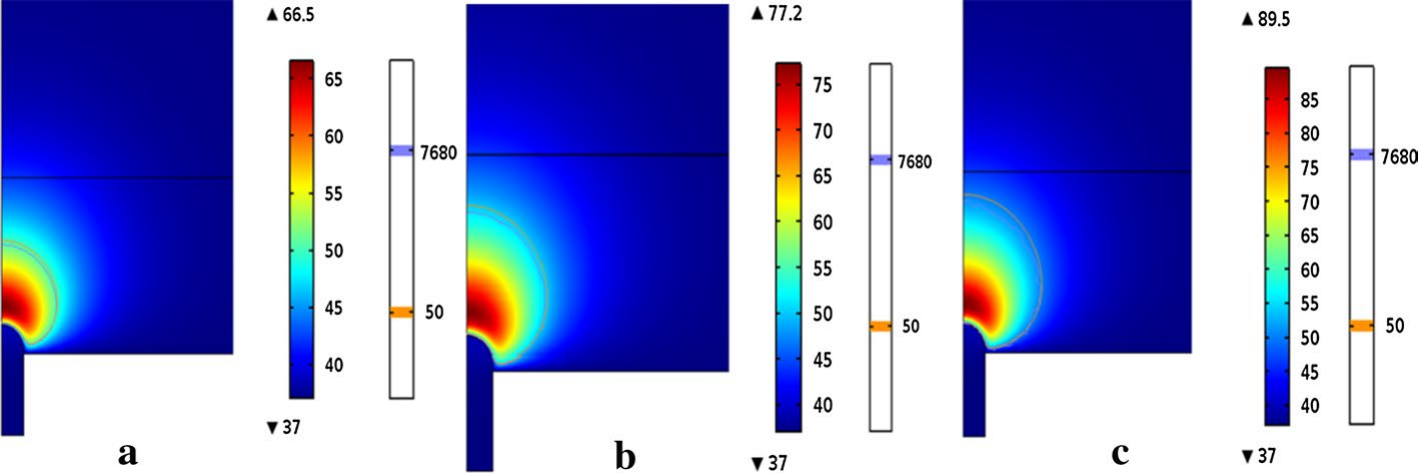
The temperature field of the atrial and the lesion outline determined by CEM43 (7680) and 50 °C isotherm (50) at voltages of **a** 30 V, **b** 35 V and **c** 40 V

Figure 3 (the temperature field of the atrial and the lesion outline determined by CEM43 (7680) and 50 °C isotherm (50) at voltages of 30, 35 and 40 V) shows the temperature distribution of the atrial tissue at the ablation time 120 s when the voltages was 30, 35 and 40 V respectively. The highest temperature was correspondingly 66.5, 77.2 and 89.5 °C respectively. The temperature field distribution appeared approximately as a semicircle which located in the myocardium tissue. Because of the blood flow the temperature in the blood was hardly rinsing at all the three voltages.

We can see from Fig. 3 that the lesion outline determined by the CEM43 °C was a little smaller than which decided by the 50 °C isotherm. And with the increasing of the voltage, the lesion range and the highest temperature of the two methods increasing. Under the same ablation time, the highest temperature rose about 11 °C with each additional 5 V.

### The comparison of the lesion outline at different times

**Fig. 4:**
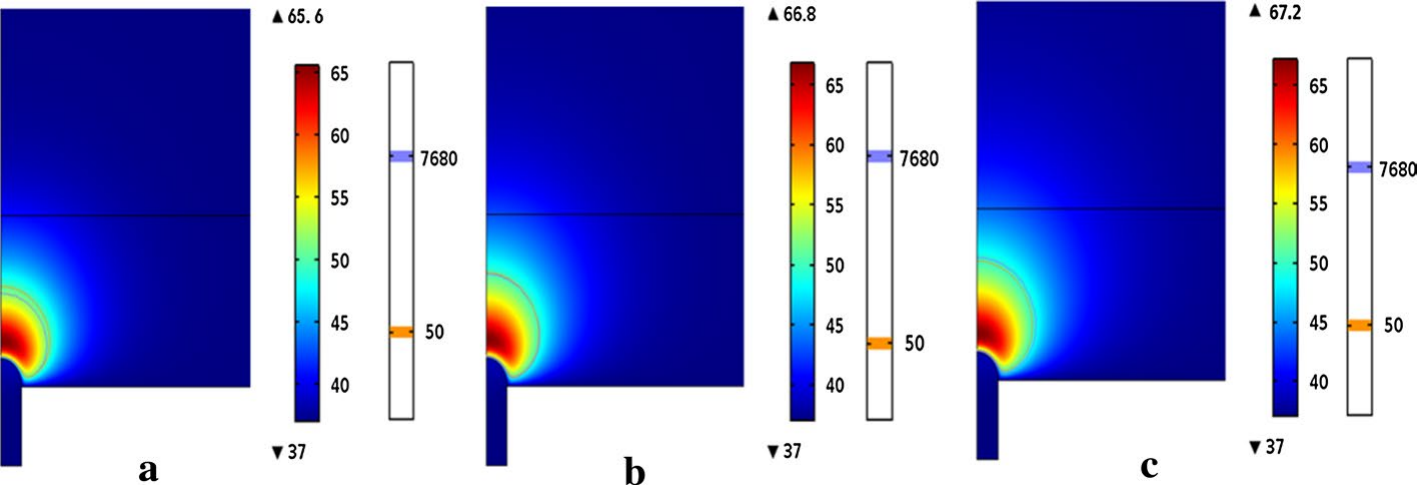
The temperature field of the atrial and the lesion outline determined by CEM43 (7680) and 50 °C isotherm (50) at time of **a** 80 s, **b** 160 s and **c** 240 s

Figure 4 (The temperature field of the atrial and the lesion outline determined by CEM43 (7680) and 50 °C isotherm (50) at time of 80, 160 and 240 s) shows the temperature distribution of the heart tissue with the voltage 30 V when the ablation time was 80, 160 and 240 s respectively. The highest temperatures was correspondingly 65.6, 66.8 and 67.2 °C respectively. The lesion outline determined by the CEM43 °C was a little smaller than which decided by the 50 °C isotherm when the ablation time was 80 s. With the increase of the time, the lesion range determined by the CEM43 °C was increasing with the larger magnitude than 50 °C isotherm. So when 160 s the isotherms overlapped and CEM43 °C isotherm was a little larger than 50 °C isotherm.

The lesion outline of both methods and the highest temperature increased with the increasing of the time. At the same voltage, the highest temperature rose about 1 °C with every additional 80 s.

### The comparison of lesion size determined by isotherm and thermal dosage

Tables 2 and 3 shows the lesion diameters at the ablative time of 60 s and 120 s while the voltage 30, 35, 40, 50 and 60 V. It can be seen from the data that the temperature distribution determined by the 50 °C isotherm predicted large. At the time of 60 s, the overestimate rate was from 12.37 to 24.9%. In Table 3, the overestimate rate was from 3.06 to 10.26%.

**Table 2:** The comparison of lesion size determined by the 50 °C isotherm and the CEM43 °C at t = 60 s

Voltage (V)	Lesion size of 50 °C (mm^2^)	Lesion size of CEM43 (mm^2^)	Overestimate rate (%)
30	9.4487	7.0958	24.9
35	14.084	11.516	18.23
40	19.091	15.619	18.18
50	28.417	23.637	16.82
60	35.568	31.168	12.37

**Table 3:** The comparison of lesion size determined by the 50 °C isotherm and the CEM43 °C at t = 120 s

Voltage (V)	Lesion size of 50 °C (mm^2^)	Lesion size of CEM43 (mm^2^)	Overestimate rate (%)
30	11.294	10.948	3.06
35	17.973	16.128	10.26
40	22.377	24.58	8.96
50	37.464	34.473	7.98
60	45.091	43.061	4.5

### The comparison of the temperature field determined by Pennes’ bioheat transfer equation and Hyperbolic equation

**Fig. 5:**
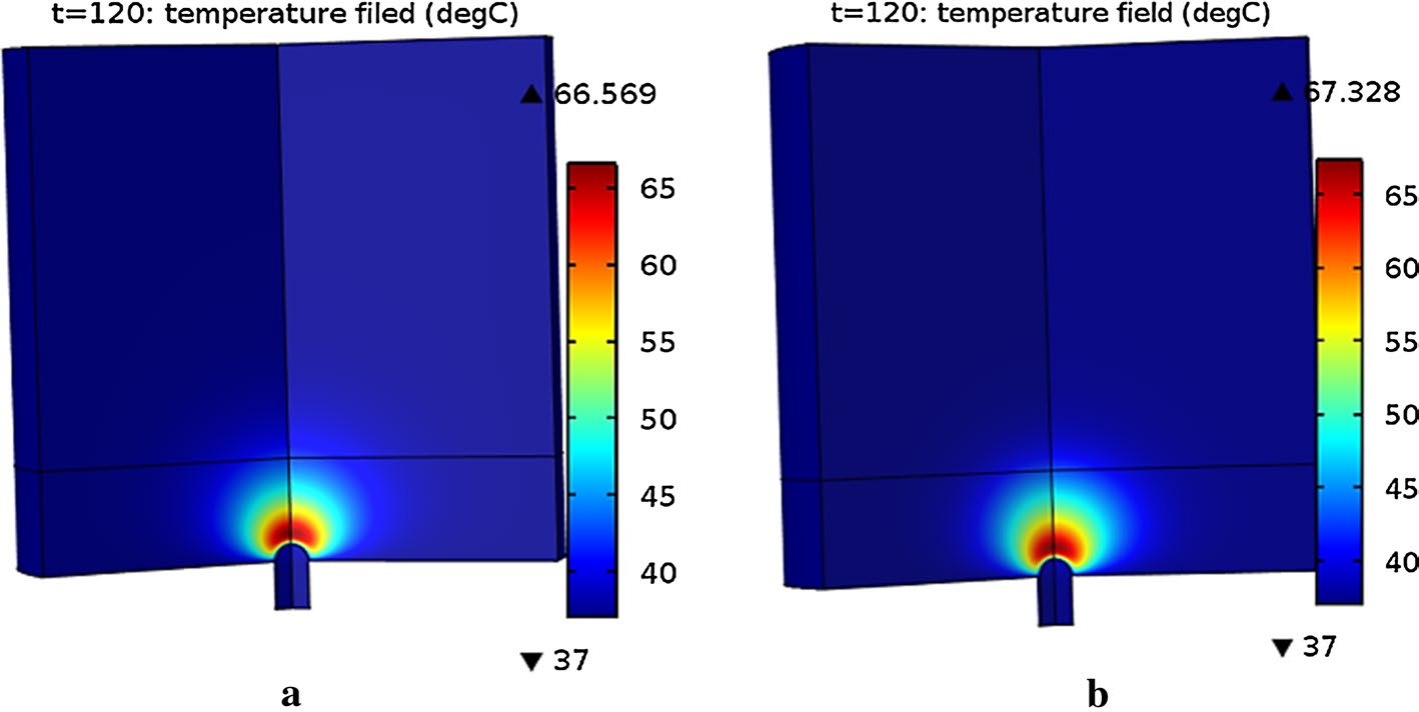
The temperature field of the atrial tissue determined by **a** Pennes’ bioheat transfer equation and **b** Hyperbolic equation

Figure 5 (The temperature field of the atrial tissue determined by Pennes’ bioheat and Hyperbolic) shows the temperature field of the heart tissue at 30 V when the ablation time was 120 s. Figure 5a shows the temperature field determined by Pennes’ bioheat transfer equation while Fig. 5b shows the temperature field determined by Hyperbolic equation. The shapes of the temperature field of the both figures were similar while the highest temperature which determined by the Hyperbolic equation was a little higher (about 0.7 °C).

**Fig. 6:**
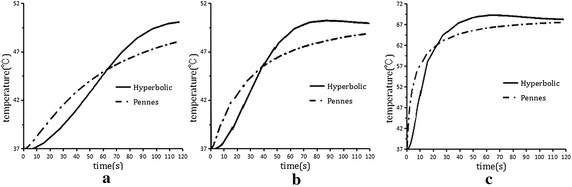
The temperature contrast curve of some points. **a** Point A. **b** Point B. **c** Point C

Figure 6 (The temperature contrast curve of some points) shows the temperature contrast of Pennes equation and Hyperbolic equation. In the start of all the three points, the temperature which determined by Pennes equation was higher than the temperature determined by Hyperbolic equation. Point A, B and C has been shown in Fig. 1. These points are set at the distance from antenna tip 0.7, 2.7 and 4.7 mm in the myocardium. The temperature curves of point A, point B and point C intersected respectively in about 65, 40 and 22 s. After intersection the temperature determined by Hyperbolic equation was higher than that temperature determined by Hyperbolic equation. And with the increasing of the time the temperature rose slowly. Curves of point B and point C shut down at the end.

**Fig. 7:**
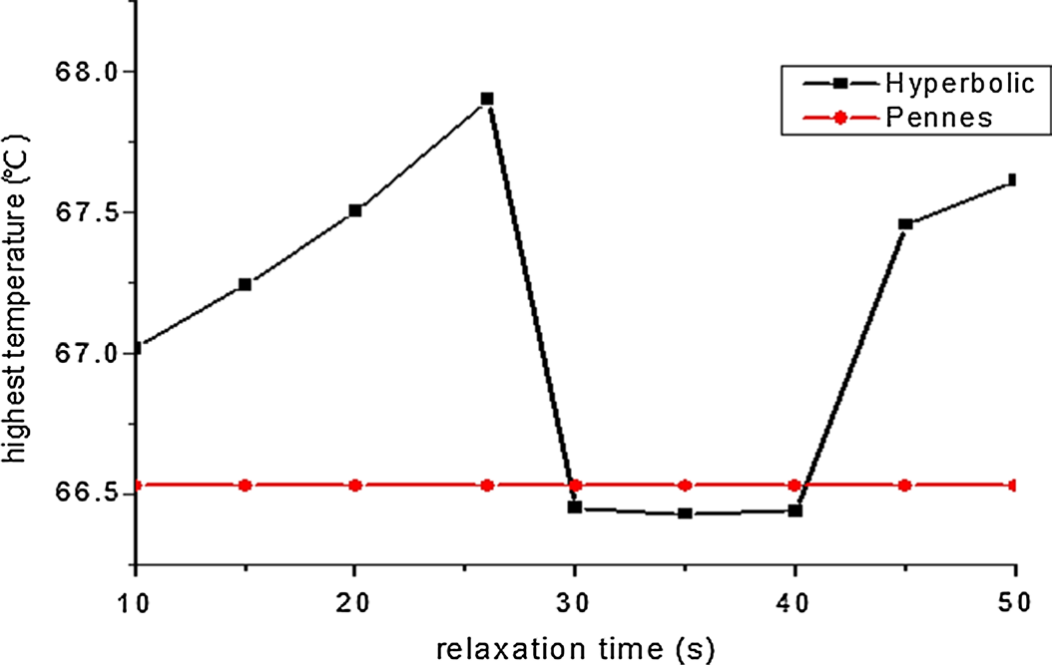
The change of the maximum temperature in different relaxation time

As shown in Fig. 7 (The change of the maximum temperature in different relaxation time), the highest temperature changing over the relaxation time. The highest temperature was a fixed value because Pennes’ bioheat transfer equation had no contact with the relaxation time. While the highest temperature determined by the Hyperbolic equation was increasing gradually with the increasing of the relaxation time within 25 s. When the relaxation time between 25 and 30 s, the highest temperature dropped rapidly. It’s relatively stable when the relaxation time between 30 and 40 s. The highest temperature increased with the increasing of relaxation time, when it was more than 40 s. And the highest temperature determined by the Hyperbolic equation is higher than determined by the Pennes’ bioheat transfer equation except the relaxation time between 30 and 40 s.

## Discussion

Due to the blood flow, the temperature in the blood can hardly raise, the temperature field looks like a semicircle in the myocardium. In above figures, it can be known the temperature increase with both the increase of voltage and the time, and the temperature increase with voltage is more obviously than the increase with time.

In this study, we used 50 °C isotherm and thermal dosage to analyze the lesion outline, and both of them increased with the increase of the voltage and time. From results we can see that the lesion outline of the thermal dosage is smaller than the lesions outline of the 50 °C isotherm in all the time when the voltage increased. The lesion range of CEM43 is smaller than the lesion range of the 50 °C isotherm when the time within 160 s.

In cardiac ablation, the duration time is 60 s to 120 s, and the voltage is 30–60 V in clinical currently. Since we compared the lesion size when the time was 60 and 120 s and the voltage 30–60 V, the size of 50 °C isotherm were larger than the size of the CEM43. Some studies researched in ex vivo liver [31] and found the two methods give similar results, but the critical isotherm was chosen; and others found that the isotherm overestimate 4.8% for the final lesion diameter than the critical CEM43 °C [8] since the RF has been shut down. Therefore the thermal dose gets more precise data.

We contrasted the temperature field determined by Pennes’ bioheat transfer equation and Hyperbolic equation. And in this study, we assumed the relaxation time was 16 s to analyze at first. And the results showed the shape of the temperature distribution similarly. We also chose 10 values from the relaxation time from 10 to 50 s to simulate, and we found that only the maximum temperature which determined by the Hyperbolic equation is little higher in addition to the relaxation time between 30 and 40 s. The temperature determined by the Hyperbolic equation was smaller than which determined by the Pennes equation in the start and with the growth of the time it become larger than the temperature of Pennes. The highest temperature determined by the Hyperbolic equation changes up and down in a small range which is smaller than 2 °C.

In pre-ablation, due to the theory of the Hyperbolic considers the limitation of the transfer of the energy heat, at the beginning of the ablation, the heat that produced by the myocardium cannot deliver in the layer and transfer through the surface blood convection. Thus the lesion area was smaller than the Pennes transfer method. As the ablation time was over the relaxation time, the electric field produced more heat, and the heat is trapped inside the myocardium, so the area was bigger than the Pennes in post ablation.

## Conclusions

Compared to 50 °C isotherm, CEM43 °C considers the temperature history, it can be more accuracy to describe the change of temperature. And from above we can know that the 50 °C isotherm overestimate the thermal dose when the ablative time within 160 s. Now the cardiac ablation time is 60–120 s, so use thermal dose to analyze can get more accurate data and its necessary for treatment.

Pennes’ bioheat transfer equation does not consider the speed of the heat while Hyperbolic equation take it into account, and the Hyperbolic method reflects the influence of heat propagation speed. In this study, we contrasted the maximum temperature in different relaxation time and found that the highest temperature changing over it. The result of the Hyperbolic is more close to the actual situation of cardiac radiofrequency ablation, so the latter can describe temperature more exactly in cardiac ablation.

There are still some limits in this research. As lack of a time-varying blood perfusion data in clinic, we did not consider this condition into the simulation. In future, we’ll take this condition into account and to get more accuracy data to compare the results.
